# ER stress, autophagy, and RNA viruses

**DOI:** 10.3389/fmicb.2014.00388

**Published:** 2014-08-05

**Authors:** Jia-Rong Jheng, Jin-Yuan Ho, Jim-Tong Horng

**Affiliations:** ^1^Department of Biochemistry and Molecular Biology, College of Medicine, Chang Gung UniversityKweishan, Taiwan; ^2^Research Center for Emerging Viral Infections, Chang Gung UniversityKweishan, Taiwan; ^3^Department of Medical Research, Chang Gung Memorial HospitalKweishan, Taiwan

**Keywords:** ATF6, eIF2α, enterovirus 71, ER stress, IRE1, unfolded protein response

## Abstract

Endoplasmic reticulum (ER) stress is a general term for representing the pathway by which various stimuli affect ER functions. ER stress induces the evolutionarily conserved signaling pathways, called the unfolded protein response (UPR), which compromises the stimulus and then determines whether the cell survives or dies. In recent years, ongoing research has suggested that these pathways may be linked to the autophagic response, which plays a key role in the cell's response to various stressors. Autophagy performs a self-digestion function, and its activation protects cells against certain pathogens. However, the link between the UPR and autophagy may be more complicated. These two systems may act dependently, or the induction of one system may interfere with the other. Experimental studies have found that different viruses modulate these mechanisms to allow them to escape the host immune response or, worse, to exploit the host's defense to their advantage; thus, this topic is a critical area in antiviral research. In this review, we summarize the current knowledge about how RNA viruses, including influenza virus, poliovirus, coxsackievirus, enterovirus 71, Japanese encephalitis virus, hepatitis C virus, and dengue virus, regulate these processes. We also discuss recent discoveries and how these will produce novel strategies for antiviral treatment.

## Introduction

The endoplasmic reticulum (ER) is a eukaryotic organelle in which an array of cell functions takes place. These include the transportation of cellular materials, provision of increased surface area for cellular reactions, and the production of proteins, steroids, and lipids. The ER may be overloaded with molecular chaperones, folding enzymes, and massive protein products during normal processes, such as in the differentiation of B lymphocytes into antibody-secreting plasma cells (Shaffer et al., 2004; Ma et al., 2010) or in highly specialized cells for secretion (Harding and Ron, 2002). In addition, dysfunction of the ER, known as ER stress, results from pathogenic stress signals, such as hypoxia (Koumenis, 2006), ER–Ca^2+^ depletion, viral infections, or agents that affect Ca^2+^ balance (i.e., thapsigargin), protein glycosylation (i.e., tunicamycin), and ER–Golgi vesicular transport (i.e., brefeldin A), which lead to accumulation of misfolded and unfolded proteins (Kaufman, 1999). To reduce the adverse effects of accumulating misfolded or unfolded proteins, the cell operates an adaptive response known as the unfolded protein response (UPR) to reduce the load of newly synthesized proteins within the ER and eliminate inappropriately folded proteins through upregulation of ER chaperone expression. In addition, proteins that fail to correctly fold are then deployed to the distal secretory pathway from the ER by the ER-associated protein degradation (ERAD) pathway of the UPR (Hampton, 2000; Yoshida et al., 2003).

There are two ERAD models for protein degradation: ubiquitin-proteasome ERAD, designated as ERAD (I), and autophagy-lysosome ERAD, designated as ERAD (II) (Fujita et al., 2007; Korolchuk et al., 2010). Both models depend on retrotranslocation of ERAD substrates from the ER back to the cytoplasm with the help of the Cdc48p–p97 complex. Most soluble misfolded proteins are cleared through the ubiquitin-proteasome system, which involves action of a cascade of three canonical ubiquitin enzymes: E1 ubiquitin-activating enzyme initiates the reaction by using ATP to covalently activate and then conjugate the ubiquitin to an E2 ubiquitin-conjugating enzyme. Ubiquitin is then transferred from the ubiquitin-charged E2 to the lysine residue of a specific target or a growing ubiquitin chain by E3 ubiquitin ligase, which results in a multiubiquitin chain-tagged substrate. Proteins that are ubiquitinated with K48-linked chains are specifically recognized by the 26S proteasome and subjected to degradation (Hershko et al., 1983). In contrast, ERAD (II) degrades both soluble and insoluble misfolded protein aggregates in autolysosome. Autophagy receptors and adaptors, called p62/SQSTM1, NBR1, HDAC6, and ALFY, bind to proteins with K63-specific monoubiquitination or polyubiquitin chains and then guide them to the concave side of developing autophagosomes (Behrends and Fulda, 2012). Notably, p62 also recognizes K48 polyubiquitin-tagged proteins for autophagic clearance upon proteasome dysfunction. In addition to the protective role of UPR, prolonged and/or excess ER stress typically activates caspase-12, an ER-resident caspase, leading to UPR-mediated cell death (Szegezdi et al., 2006).

**Figure 1:**
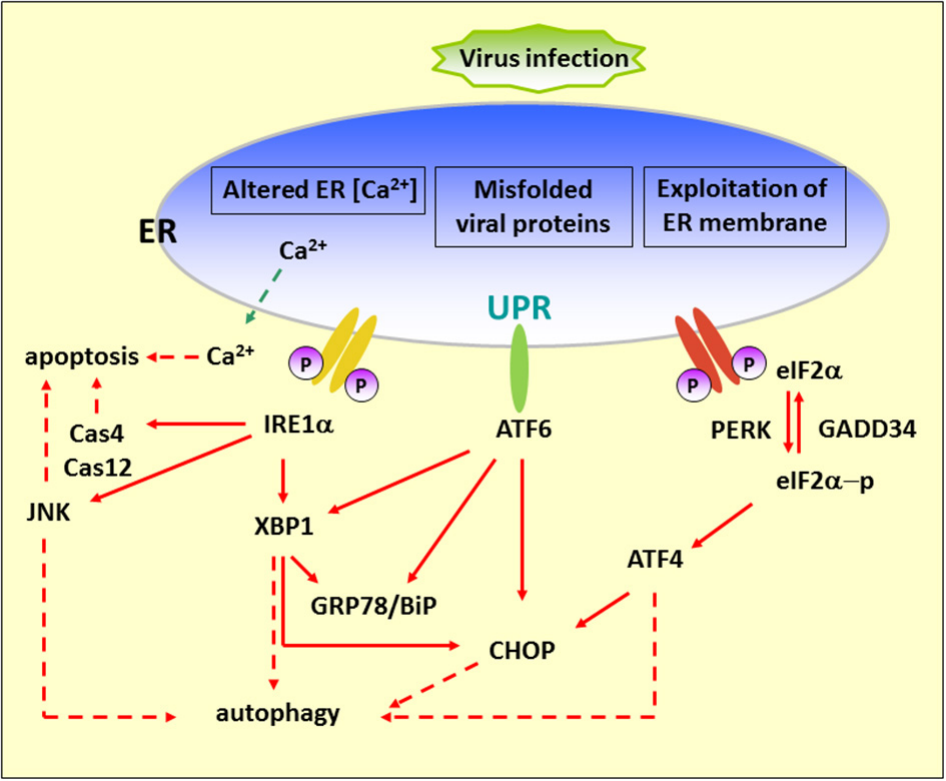
**Diagram of the UPR arms and their connection to autophagy**. Alteration of ER functions results from stress signals by RNA virus infection, by the exploitation of ER membrane for viral replication, rapid accumulation of viral proteins, imbalance of calcium concentration by viroporin, and the sabotage or depletion of ER membrane for viral release. This leads to the accumulation of misfolded and unfolded proteins, which triggers ER stress. To alleviate this adverse effect, the cells operate an adaptive UPR to reduce the load of the newly synthesized proteins in the ER by activating the PERK–eIF2α branch and eliminating inappropriate protein accumulation by upregulating ER chaperone proteins through IRE1 and ATF6 branches. In addition, the incurable misfolded proteins undergo retrotranslocation from the ER into cytosol for degradation by an ERAD mechanism. ER stress can contribute to autophagy via activation of JNK, XBP1, CHOP, and ATF4. Red dash arrows indicate the final outcome of the activated pathways, such as apoptosis and autophagy, caused by viral infection. Red solid arrows indicate the UPR pathways.

Basal autophagy plays a key role in maintaining cellular homeostasis through eliminating unwanted proteins and damaged organelles by cellular self-digestion in the lysosome to fulfill the demand for the building blocks required for cell survival (Levine and Klionsky, 2004; Shintani and Klionsky, 2004). Recently, the study of autophagy regulation has grown in different research areas, including regulation of cancer development and progression (Mahoney et al., 2013a), lipid metabolism (Singh et al., 2009), degenerative diseases (Wang et al., 2006), and the control of viral pathogenesis (Jackson et al., 2005). The first step of autophagy relies on the formation of an isolation membrane at the so-called preautophagosomal site (PAS) where a system of evolutionarily conserved proteins (Atg proteins) comes together. Recent reports have revealed that the ER serves as a subcellular platform for autophagy initiation (Axe et al., 2008). The elongation of the initial autophagic membrane requires continued processing by two ubiquitin-like protein-conjugation systems, the Atg12 and LC3 systems, which modify the autophagy proteins, Atg5 and Atg8/LC3, respectively (Geng and Klionsky, 2008). The autophagosome then fuses with endosomal and/or lysosomal vesicles to create an autolysosome, where digestion of intracellular components occurs (Eskelinen, 2005). In addition, autophagy can be induced by various physiological and pathological conditions such as nutrient deprivation, oxidative stress, and pathogen infections. The live-or-dead signal is modulated by UPR and autophagy and several lines of evidence suggest there is communication between these two pathways (Bernales et al., 2006; Ogata et al., 2006; Yorimitsu et al., 2006; Salazar et al., 2009); thus, it is believed that these two pathways could be a therapeutic target in certain circumstances (Figure 1). Herein, we review recent findings, focusing on the regulation of the UPR and autophagy involved in RNA virus infection as a new antiviral strategy.

## How RNA virus infection causes ER stress

Viral virulence is determined by successful entrance, replication in the host cell, and release of mature virion. During the life cycle, ER stress may arise from the exploitation of the ER membrane, accumulation of misfolded proteins, imbalance of calcium concentration by viroporin, and the sabotage or depletion of the ER membrane during virion release. Details of viral effects are given as follows.

### Exploitation of ER membranes

Many positive-strand RNA viruses cause the rearrangement of host intracellular membrane compartments that house replication complexes. ER, *trans*-Golgi, or lysosomes are the likely origin of virally induced membranes (Miller and Krijnse-Locker, 2008; Korolchuk et al., 2010). Upon poliovirus (PV) and coxsackievirus B3 (CVB3) infection, clusters of vesicles have been considered to derive from ER, although other cellular compartment marker proteins also colocalized with viral nonstructural proteins (Schlegel et al., 1996; Van Kuppeveld et al., 1997). Consistent with these findings, our previous study indicates that enterovirus 71 (EV71) nonstructural 2C protein, which participates in viral replication, is associated with the ER membrane through direct interaction with ER membrane protein reticulon 3 (RTN3), which is required and sufficient for immediate early virus replication and translation (Tang et al., 2007). In the RTN3 siRNA knockdown cells, synthesis of the 2C protein was ablated. However, in the RTN3 rescue cell line 2A3, the synthesis of viral protein and RNA was restored. Moreover, the interactions between RTN3 and two EV71 2C homologs of PV and CVA16 have been confirmed (Tang et al., 2007). Immunofluorescence studies reveal that replication of *Flaviviruses* dengue virus (DENV) and hepatitis C virus (HCV) may take place on perinuclear ER membranes (El-Hage and Luo, 2003). DENV2 nonstructural protein 2 (NS2A) is a 22-kDa hydrophobic protein containing five integral transmembrane segments that span the ER membrane. Functional analysis reveals that NS2A involves both DENV RNA synthesis and virion assembly/maturation (Xie et al., 2013). Furthermore, DENV infection induces ROCK-dependent vimentin rearrangement and subsequent ER redistribution (Lei et al., 2013). In addition, the HCV ER integral membrane protein, NS4B, is responsible for rearranging the ER membrane and inducing the formation of new ER-derived membrane structures, and this is possibly negatively regulated by RTN3-NS4B interaction (Lundin et al., 2003; Wu et al., 2014).

### Interference with host protein glycosylation by viruses

The N-glycosylation pathway in the ER modifies a mass of proteins at the asparagine residue of the consensus sequence Asn-X-Ser/Thr, where X is any amino acid except Pro (Kornfeld and Kornfeld, 1985; Gavel and Von Heijne, 1990). The modification influences protein folding and attributes various functional properties to the protein. Thus, interference with host protein glycosylation by viral proteins competing for the modification process may cause ER stress.

Viruses, including influenza A virus (IAV), hepatitis virus, and Japanese encephalitis virus (JEV), use this host cell process to enhance viral pathogenesis through facilitating folding and trafficking, affecting receptor interaction, and modulating host immune responses (Tatu et al., 1995; Dubuisson and Rice, 1996; Zai et al., 2013). Hemagglutinin (HA) of IAV is a type I transmembrane glycoprotein that determines viral antigenicity. Throughout the glycosylation process, HA rapidly associates with calnexin in a monoglucosylated form. Once folded, the HA monomers dissociate from calnexin and assemble into trimeric structures in the ER or in the intermediate compartment (Tatu et al., 1995). HCV envelope glycoproteins E1 and E2 have been shown to cooperate for the formation of a functional noncovalent heterodimer (Dubuisson et al., 1994; Dubuisson and Rice, 1996). Based on studies of HCV pseudoparticles, coexpression of both envelope glycoproteins has been shown to be necessary to produce infectious pseudoparticles (Bartosch et al., 2003). Glycosylation also occurs in JEV and WNV proteins, namely the precursor of membrane protein (prM), the envelope protein (E), and the nonstructural protein NS1, which affects the efficiency of virus release and infection (Hanna et al., 2005; Zai et al., 2013).

### Viroporins

Typically, viroporins are composed by integral membrane proteins to form a hydrophilic pore, which targets different cellular compartments and ions, thus affecting various viral functions (Nieva et al., 2012). For example, IAV M2 reduces the acidity of vesicular compartments to trigger virus uncoating. It is also required for viral assembly and release. In the case of ER-targeting viroporins, rotavirus-encoded NSP4 modifies the calcium homeostasis by enhancing the calcium permeability of the ER membrane. This may be associated with virus-induced cell death and subsequent release of NSP4, which in turn causes activation of the phospholipase C-IP3 cascade in neighboring noninfected cells and is responsible for viral pathogenesis (Tian et al., 1995, 1996; Dong et al., 1997). On the other hand, 2B proteins of picornaviruses also participate in the remodeling of membrane structures and the formation of replication complexes (De Jong et al., 2008). Among them, CBV3 2B, PV1, and rhinovirus 2B are present at the membranes of the ER and Golgi complex and are responsible for the release of Ca^2+^ and H^+^ from these organelles.

### Virion budding

Rotavirus studies propose that the double-layered particle (DLP)–VP4–NSP4 complex breaches the ER membrane and penetrates into the ER. The viral capsid protein, VP7, re-envelopes the immature particle (DLP) after removal of the ER membrane and NSP4, and forms the infectious triple-layered particle (Tian et al., 1996; Trask et al., 2012).

## Regulation of UPR by viruses

The induction of individual branches or part of the UPR by viruses was reported previously. Viruses have also evolved different means to modulate the arms of the UPR, which consequently expanded both the temporal and spatial superiority for virus replication or completion of the life cycle.

### eIF2α pathway

**Figure 2:**
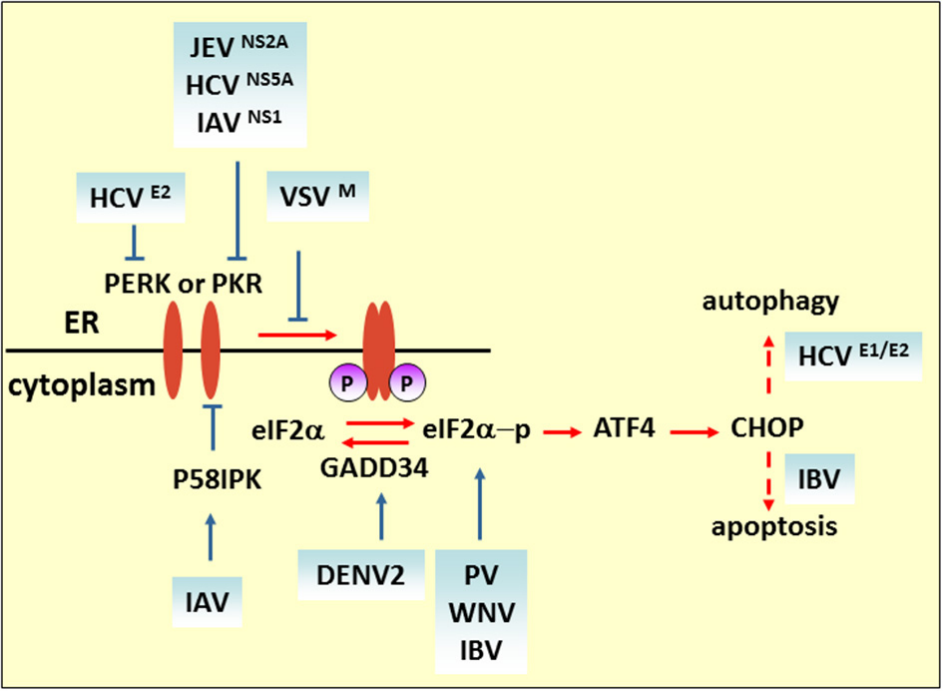
**eIF2 pathway under viral infection**. The M protein of VSV, the E2 and NS5A proteins of HCV, and NS2A of JEV counteract the phosphorylation of eIF2α for viral replication. Blue solid arrows indicate the direct target of the virus or viral proteins. IAV also targets eIF2α by inducing P58IPK, a cellular inhibitor of PERK and PKR. IBV upregulates eIF2α–ATF4–CHOP-mediated apoptosis to benefit viral replication.

It has been reported that viruses regulate the host translational machinery to promote viral protein synthesis by inhibiting the synthesis of proteins involved in host immune responses. In enteroviruses, 2A and 3C proteases target translation factors such as eIF4GI and poly(A)-binding protein (PABP) to impede host translation (Lloyd, 2006). Moreover, modulation of the integrated stress response (ISR), which is determined by phosphorylation of eIF2α to attenuate cellular translation, is another strategy for promoting virulence (Figure 2) (Sonenberg and Hinnebusch, 2009). Four eIF2α kinases have been identified: heme-regulated inhibitor (HRI), which is a response to heme deficiency (Chen, 2007); double-stranded RNA-dependent protein kinase (PKR), which is induced by interferon (IFN) and activated by double-stranded RNA (dsRNA) during viral infection (Meurs et al., 1990); general control nonderepressible-2 (GCN2), which is activated by serum and amino acid deprivation (Harding et al., 2000); and finally, PKR-like ER kinase (PERK or PEK), which is activated by unfolded proteins in the ER (Ron, 2002).

Some researchers consider that eIF2α phosphorylation plays a role in hampering viral protein synthesis. For example, upon VSV infection, the induction of activated PERK only correlates with eIF2α phosphorylation at the later stage of infection. In MEF cells carrying a phosphorylation-insensitive eIF2α S51A variant, viral protein synthesis increased compared with a wild-type control, indicating that eIF2 phosphorylation is inhibitory to viral protein synthesis. As demonstrated by matrix (M) protein mutant virus (rM51RM), a viral protein (M protein) is involved in counteracting the antiviral response of the phosphorylation of eIF2α (Connor and Lyles, 2005). Like VSV infection, Chikungunya virus (CHIKV) induces PERK activation but delays eIF2α phosphorylation. The expression of CHIKV NSP4, which is the RNA-dependent-RNA polymerase, contributed to suppression of eIF2α phosphorylation, thus ensuring translation of viral proteins (Rathore et al., 2013). Furthermore, viruses containing type I or type II internal ribosomal entry sites (IRESs), such as PV, foot-and-mouth disease virus, mengovirus and EMCV, require many canonical translation initiation factors for initial replication (Beales et al., 2003; Sarnow, 2003). It is reported that PV switches translation mode from an eIF2-dependent to an eIF2-independent one during the course of infection to ensure efficient proliferation. Furthermore, studies have shown that the C terminal of the eIF5B fragment, cleavage by 3C proteases, and proteolytic activity of 2A^pro^ can stimulate virus IRES translation of enteroviruses (De Breyne et al., 2008; Redondo et al., 2011). Interestingly, it is reported that phosphorylation of eIF2α is required for activation of IRES during cell differentiation (Gerlitz et al., 2002). Thus, whether the phosphorylation level of eIF2α positively correlates with IRES-dependent viral mRNA translational efficiency remains to be determined. Some viruses regulate the eIF2α pathway by interfering with the activation of eIF2α kinases. HCV NS5A protein, containing an IFN sensitivity-determining region (ISDR), interferes with PKR activity by binding to a PKR dimerization domain (PKR residues 244–296) (Gale et al., 1998), while HCV E2 protein binds to PERK and inhibits downstream eIF2α phosphorylation by acting as a pseudosubstrate (Pavio et al., 2003). Interestingly, it is reported that NS5A stimulates eIF2α phosphorylation in the absence of PKR, implying that NS5A may activate other eIF-2α kinases to regulate eIF2α phosphorylation (Tardif et al., 2002). Overexpression of HCV NS2 induces eIF2α phosphorylation (Von Dem Bussche et al., 2010). Taken together, these studies indicate that HCV proteins modulate eIF2α pathway in a complex way, and the effect of regulation on virus replication cannot be established unequivocally. The N-terminal region of NS2A of JEV contains a sequence that is highly similar to HCV NS5A ISDR and also inhibits PKR-induced eIF2α phosphorylation (Tu et al., 2012). DENV2 infection triggers and then suppresses PERK-mediated eIF2α phosphorylation by elevating the expression of growth arrest and DNA damage-inducible protein-34 (GADD34), which acts together with phosphatase 1 (PP1) to dephosphorylate eIF2α-P (Pena and Harris, 2011). Influenza virus nonstructural protein NS1 interferes with dsRNA binding to PKR, and the infection also induces and activates P58IPK, a cellular inhibitor of PKR and PERK. Both strategies deployed by NS1 and P58IPK prevent PKR dimerization and autophosphorylation, which limits eIF2α phosphorylation (Lee et al., 1992; Lu et al., 1995; Yan et al., 2002).

In some circumstances, such as when the host immune system specifically recognizes foreign viruses and kills them with cytotoxic T lymphocytes, or when cell death is directly induced in virus infected cells to prevent completion of the replication cycle, apoptotic cell death is considered to be a host strategy for fighting against viral infections. ATF4 is a transcriptional activator of the ISR, which is involved in the expression of ISR target genes such as c/EBP homologous protein (CHOP) and GADD34 (Ma and Hendershot, 2003). CHOP was originally identified as a transcriptional factor eliciting ER stress-induced apoptosis. In cells subjected to West Nile virus (WNV) infection, eIF2α phosphorylation and CHOP-mediated apoptosis were induced. Both viral protein expression level and virus titer are increased in CHOP-deficient cells (Medigeshi et al., 2007). On the other hand, a virus may induce apoptosis to facilitate replication or the spread of viral progeny. It is reported that coronavirus infectious bronchitis virus (IBV) upregulates eIF2α–ATF4–CHOP signaling in infected cells and that it relies on PERK or PKR activation. Knockdown of CHOP reduces IBV-induced apoptosis through activation of the extracellular signal-related kinase (ERK). Viral protein expression level is moderately suppressed in CHOP-knockdown cells, which suggests that upregulation of CHOP-mediated apoptosis during IBV infection probably promotes virus replication (Liao et al., 2013).

In addition to regulation of cell death, it is reported that HCV induces the expression of CHOP at mRNA and protein levels and is correlated with autophagy induction; knockdown of CHOP not only increases HCV PAMP-mediated innate immune activation, but also elevates its inhibitory effect on virus replication (Ke and Chen, 2011). However, upstream CHOP induction is a matter of debate. Overexpression of HCV E1 and/or E2 induces the expression of CHOP in a PERK-dependent manner (Chan and Egan, 2005); while upon HCV infection, CHOP protein is upregulated by PERK, activating transcription factor (ATF6), and inositol-requiring transmembrane kinase/endonuclease 1 (IRE1) collectively.

### ATF6 pathway

**Figure 3:**
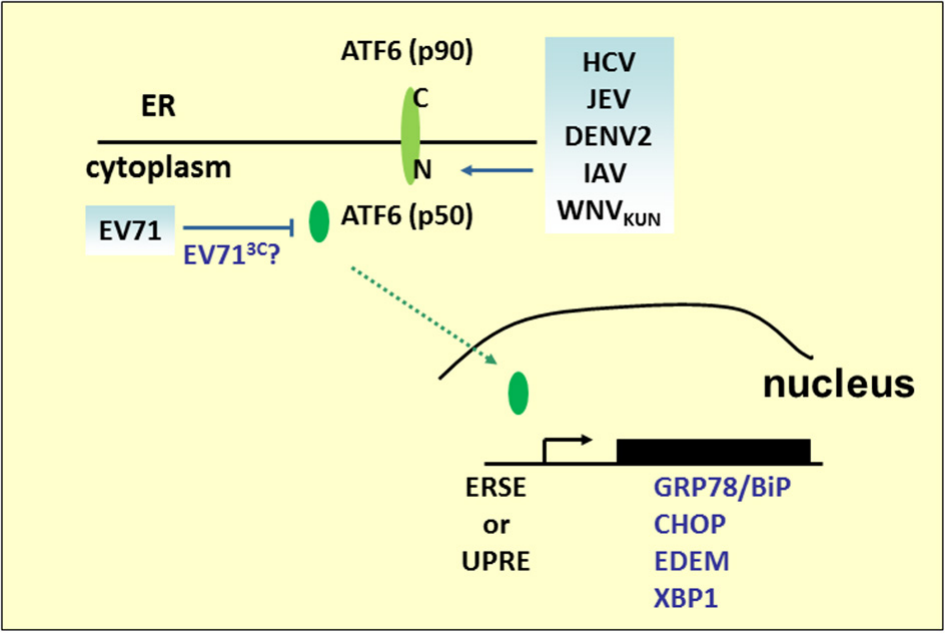
**ATF6 pathway under viral infection**. Many RNA viruses activate the UPR pathway by cleaving ATF6 to release the p50 fragment. The N-terminal p50 with transcription activity enters the nucleus to activate the expression of ER stress and ERAD genes, such as GRP78/BiP, CHOP, XBP1, or EDEM. However, the p50 fragment was not detected in the EV71 infection.

ATF6 is a type 2 transmembrane protein of 670 amino acids and is constitutively expressed as a 90-kDa protein (p90ATF6). Its C-terminal region is located in the ER, whereas the N-terminal region is located on the cytosolic side (Figure 3). Upon ER stress, ATF6 is cleaved to an N-terminal 50-kDa protein (p50ATF6) sequentially by the Golgi site-1 and site-2 proteases (S1P and S2P) (Ye et al., 2000). Nuclear translocation of p50ATF6, as a transcription factor, activates expression of ER stress and ERAD genes including ER chaperones, CHOP (*aka* GADD153), EDEM1, and X-box-binding protein 1 (XBP1) by targeting the *cis*-acting ER stress response element (ERSE) (CCAAT-N9-CCACG) and UPR element (UPRE) (GATGACGTG(T/G) NNN(A/T)T), although ATF6 has a much higher affinity for ERSE (Yoshida et al., 1998). In addition to directly regulating gene expression, ATF6 also modulates the innate immune response. Under subtilase cytotoxin (SubAB) treatment, cleavage and degradation of GRP78/BiP leads to activation of the AKT–NF-κB pathway through ATF6 activation (Yamazaki et al., 2009). Based on its pivotal role of connecting the arms of the UPR and converging the UPR and immune response, many viruses preferentially regulate ATF6 pathways to benefit replication. In WNV strain Kunjin (WNV_KUN_)-infected cells, expression of ATF6-target genes increases, but viral production decreases in ATF6 knockout MEF cells. Moreover, in ATF6 knockout MEF cells, phosphorylation of eIF2α, downstream CHOP activity, and Jak–STAT1 phosphorylation induced by IFNα are upregulated upon infection, which implies that virus-induced ATF6 activation is a prosurvival mechanism required for replication and inhibition of the antiviral signaling pathway (Ambrose and Mackenzie, 2013). However, it is still unclear whether WNV_KUN_ NS4A and NS4B, potent inducers of the UPR, inhibit IFNα-induced Jak–STAT signaling in an ATF6-dependent manner (Ambrose and Mackenzie, 2011). Other *Flavivirus* infections, including HCV, JEV, and DENV2, also induce cleavage of ATF6, nuclear translocalization of ATF6 and increases in chaperone proteins expression. In HCV replication, silencing of ATF6 reduces HCV intracellular mRNA levels (Ke and Chen, 2011). However, in JEV-infected cells, knockdown of the ATF6-targeted gene, GRP78, by siRNA did not affect JEV viral RNA replication, although it did impair virus assembly or release. In sucrose gradient, mature JEV viruses that do not cofractionate with GPR78 displayed a significant decrease in viral infectivity, indicating that JEV acts with GPR78 to promote its infectivity (Wu et al., 2011). Notably, DENV2 triggers ATF6 signaling in a cell-type-specific manner. In A549 cells, nuclear-localized ATF6 was observed (Umareddy et al., 2007); however, no activating events can be detected in human fibrosarcoma 2fTGH cells, therefore, GPR78 upregulation may be mediated in an ATF6-independent fashion (Pena and Harris, 2011). This cell-type-specific regulation of ATF6, also observed in IAV infection, p50ATF6, and its target gene ERp57/GRP58 expression (Roberson et al., 2012), has been shown to increase in murine primary tracheal epithelial cells infected with influenza A/PR/8/34, which is known to be involved in influenza virus HA protein folding (Solda et al., 2006). Knockdown of ERp57 abrogates viral progeny production. However, ATF6 activity is not induced in infected human tracheobronchial epithelial (HTBE) cells (Hassan et al., 2012).

Although ATF6-mediated transcriptional activation is an ongoing research field, another role for ATF6 in virus infection has emerged. We have previously demonstrated that EV71 infection results in the decline of p90ATF6, while the GRP78 promoter containing classical ERSE sites responsive to p50ATF6 in EV71-infected cells was not activated (Jheng et al., 2010). Indeed, two potential 3C cleavage sites (glutamine–glycine; QG) located at adjacent amino acids 511–512 and 516–517 near the C terminus of p90ATF6 were computationally predicted. It would be interesting to investigate the role of viral 3C in the regulation of ATF6, for its possible contribution in manipulating virus infection.

### IRE1 pathway

IRE1 is an ER-localized type I transmembrane protein containing an ER luminal dimerization domain and cytosolic kinase and RNase domains (Mori et al., 1993; Sidrauski and Walter, 1997). During ER stress, accumulation of unfolded proteins in the ER stimulates IRE1 oligomerization and autophosphorylation (Figure 1). Its endoribonuclease activity initiates an unconventional splicing of the XBP1 mRNA, excising a 26-nt sequence and shifts the reading frame to produce a functional isoform XBP1(S), which contains a C-terminal transactivation domain absent from the unspliced form, XBP1(U). XBP1(S) then translocates to the nucleus where it induces expression of target genes containing UPRE or ERSE. These target genes are involved in ERAD, chaperone protein production, and ER membrane biosynthesis (Shamu and Walter, 1996; Friedlander et al., 2000).

**Figure 4:**
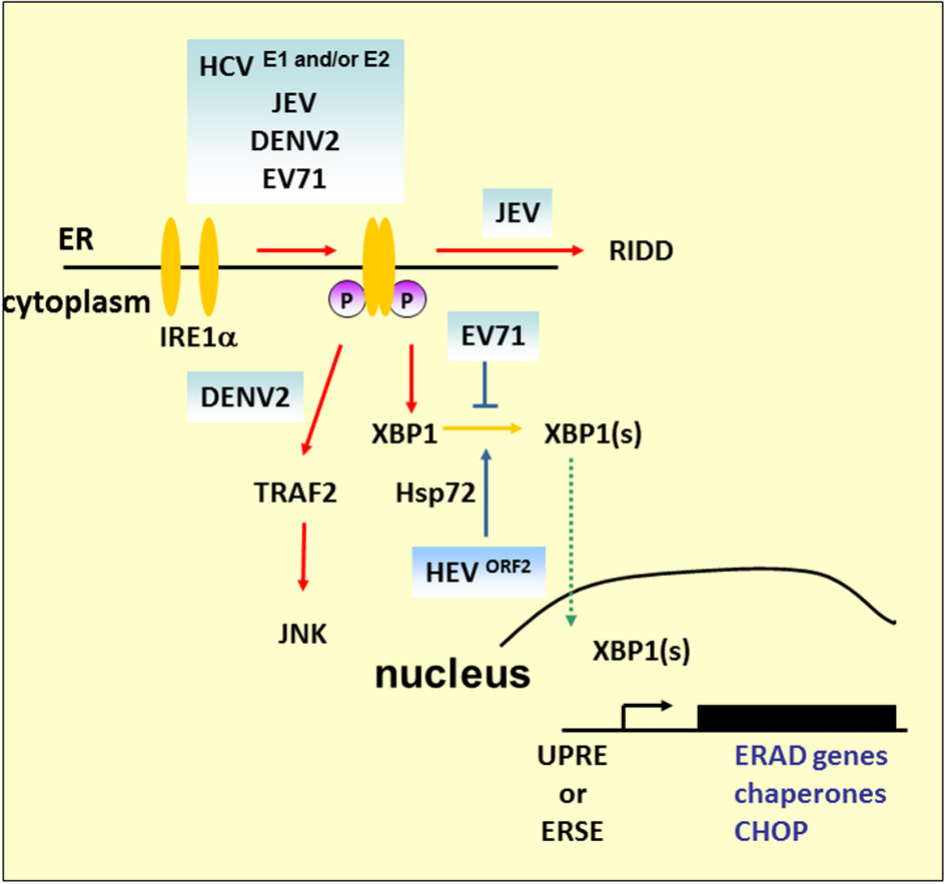
**IRE1 pathway under viral infection**. In addition to mediating Xbp1 mRNA splicing, studies demonstrated that Ire1 activates RIDD to promote the degradation of mRNAs encoding ER-targeted proteins to reduce the load of ER client proteins during ER stress. The mammalian IRE1–TRAF2–JNK pathway, independent of XBP1 splicing, may lead to the activation of apoptosis after prolonged ER stress. HCV and its structural proteins E1 and E2 play an important role in the activation of the IRE1–XBP1–ERAD pathway. Overexpression of ORF2 of HEV can upregulate antiapoptotic protein Hsp72 to activate XBP1 splicing. However, further study is required to determine whether HEV infection can activate XBP1 via Hsp72. DENV2 infection activates CHOP and GADD34 expression downstream of IRE1–XBP1 signaling. However, apoptosis activation by JNK, but not CHOP, is essential for DENV2 infection.

Studies of the IRE1 signaling pathway demonstrate its significant role in virus infection (Figure 4). HCV glycoprotein E2 is an example of a virus-derived ERAD substrate. HCV infection activates the IRE1–XBP1–EDEM pathway, where EDEM1 and EDEM3, but not EDEM2, interact with HCV E2 to accelerate its degradation. Either knockdown of EDEMs or treating cells with kifunensine (KIF), a potent inhibitor of ER mannosidase, interferes with the binding of EDEMs with SEL1L, a component of ERAD complex, stabilizes E2 expression, and enhances virus replication and viral particle production. However, there is no interaction between EDEM proteins and the JEV envelope protein and abolishing the ERAD pathway by KIF does not affect JEV production (Saeed et al., 2011). The results emphasize the pivotal role of the ERAD pathway in the life cycle of specific viruses. Interestingly, UPRE reporter activity or ERAD of misfolded null Hong Kong α-antitrypsin is reduced in cells carrying HCV replicons, which lack structural proteins, even though upstream XBP1 splicing occurs (Tardif et al., 2004). This implies that HCV structural proteins play a key role in XBP1-mediated UPRE activation, and this is supported by a related study demonstrating that HCV E1 and/or E2 activates the XBP1–ERAD pathway (Chan and Egan, 2005). Furthermore, the IRE1 signaling pathway also participates in viral protein retrotranslocation. Hepatitis E virus (HEV) ORF2 is an N-linked glycoprotein which is cotranslationally translocated into the ER while a significant fraction of it is also observed in the cytoplasm. Based on the results of tunicamycin and KIF treatment, it is believed that glycosylation and ERAD are essential for ORF2 retrotranslocation from the ER to the cytoplasm (Surjit et al., 2007). However, no ubiquitination of ORF2 can be observed, and retrotranslocated ORF2 protein was stable in the cytoplasm when the cells were treated with proteasome inhibitor MG132, which suggests that ERAD is required for ORF2 access to the cytoplasm. Microarray analysis reveals that ORF2 overexpression causes upregulation of Hsp70B, Hsp72, and Hsp40. Hsp72 is an antiapoptotic heat shock protein that directly interacts with ORF2 (John et al., 2011). It is reported that expression of Hsp72 enhances XBP1 mRNA splicing and protects cells from ER stress-induced apoptosis by association with IRE1 (Gupta et al., 2010). Thus, further investigation is needed to examine the correlations of ORF2, IRE1, and Hsp72 in HEV replication.

Under harsh ER stress, the activation of IRE1–XBP1 can also lead to the induction and expression of CHOP. DENV2 infection induces CHOP, and GADD34 expression is a downstream event of IRE1–XBP1 signaling. Of note is that induction of CHOP does not lead to apoptosis markers such as decreased expression of Bcl-2 or proteolytic cleavage of pro-caspase-9, pro-caspase-3, or PARP, which indicates a role beyond guiding cell death in infected cells (Pena and Harris, 2011). Indeed, it has been reported that CHOP exhibits protective effects against radiation-induced apoptosis or has a role in autophagy induction (Mayerhofer and Kodym, 2003; Ke and Chen, 2011). Another ER stress-induced cell death that relies on the IRE1–TRAF2 pathway is implicated in JNK activation (see Figure 4). The role of this pathway is emphasized by DENV2 infection; silencing of IRE1 decreases the virus titer, but the viral progeny output is not affected by silencing of XBP1 (Pena and Harris, 2011). However, JNK pathway inhibitors diminished virus yield significantly, which suggests that activation of JNK is essential for DENV2 infection (Ceballos-Olvera et al., 2010). Our previous findings also demonstrated that EV71 phosphorylates IRE1, but inhibits the expression of XBP1. The overexpression of XBP1 in cells appeared to inhibit viral entry, and therefore reduce viral RNA and viral particle formation (Jheng et al., 2012). As previous studies have reported that picornavirus infections induce JNK activation (Kim et al., 2004; Peng et al., 2014), further detailed studies of the IRE1-JNK activation in EV71 infection would extend our understanding of the contributions of IRE1-JNK in the virus life cycle.

IRE1 has also been linked to the mediation of the selective degradation of a subset of ER-localized mRNAs in a process known as regulated IRE1-dependent degradation (RIDD) (Hollien and Weissman, 2006). Mutation or removal of the signal sequences in targeted mRNAs prevents their decay (Kimmig et al., 2012). However, it has been observed that Drosophila mRNA Smt3, a homolog of a small ubiquitin-like modifier (*aka* SUMO), lacks any ER-targeting sequence, and is a noncanonical RIDD target, which implies that unknown specific features other than ER localization are involved in defining the RIDD substrates (Moore et al., 2013). RIDD has been suggested to play adaptive roles by reducing protein translocation load, such as decrease of proinsulin expression in pancreatic beta-cells faced with chronic high glucose, and protecting liver cells from acetaminophen-induced hepatotoxicity (Lipson et al., 2008; Hur et al., 2012). Alternatively, RIDD has also been suggested to play destructive roles under unmitigated ER stress because continued degradation of mRNAs encoding secretory cargo proteins and proteins involved in ER-resident protein folding occurs.

In addition to IRE1–XBP1 activation, JEV also induces activation of the RIDD cleavage pathway (Bhattacharyya et al., 2014). The addition of STF083010, a specific inhibitor of IRE1 RNase activity, to infected cells decreases the Tg-induced Xbp1 splicing and potential RIDD target transcripts. It also decreases viral protein expression as well as mature progeny formation, but does not affect viral RNA synthesis, which indicates that JEV viral RNA is not a substrate of RIDD, and RIDD activation is beneficial for viral infectivity.

It is not clear whether other viral infections trigger RIDD. To extrapolate from the study of HCV, HCV replicons activate the phosphorylation of IRE1 but impede XBP1 activation (Tardif et al., 2004). Depletion of IRE1 attenuates replicon translation, which implies that RIDD may enhance viral protein synthesis. Thus, the study of HCV replicon may have potential for deciphering the role of RIDD in HCV infection because it could uncouple XBP1 signaling from IRE1 activation.

## Autophagy

Autophagy is a vesicular process that results in the degradation of the sequestered component, which can then be recycled by the cell. In mammalian cells, a complete autophagy includes the following four steps. (1) Induction. Induction is initiated by activation of the Unc-51-like kinase 1 (ULK1) complex. The ULK1 complex contains ULK1, focal adhesion kinase (FAK)-family-interacting protein of 200 kD (FIP200), Atg13 and Atg101 (Mizushima, 2010). ULK1 complex activity would be, at least, modulated by mTORC1, Akt, and AMPK (Inoki et al., 2003; Bach et al., 2011; Egan et al., 2011; Kim et al., 2011). mTORC1 is a serine/threonine kinase complex, which phosphorylates ULK1 and Atg13 and also inhibits autophagy. Akt and AMP-activated protein kinase (AMPK) phosphorylate TSC2 at different residues, which results in the GTP hydrolysis of Rheb and indirectly antagonizes the mTORC1 signaling pathway. Recently, the combination of bioinformatic and proteomic approaches has identified ULK1 as a direct target of AMPK and as involved in autophagy induction. (2) Vesicle nucleation. The Beclin1–PI3KC3 complex, generating PI3P, is essential for recruitment of PI3P effectors including DFCP1, WIPIs upstream of Atg proteins and lipids recruitment to the PAS, which is required for autophagosome construction (Proikas-Cezanne et al., 2004; Axe et al., 2008). Importantly, the activity of the Beclin1–PI3KC3 complex depends on its subunit composition. Complexes containing Atg14-like protein (ATG14L or Barkor) or ultraviolet irradiation resistance-associated gene (UVRAG) activate autophagy (Itakura et al., 2008); nevertheless, the RUN domain and cysteine-rich domain containing Beclin 1-interacting protein (Rubicon) act as negative regulators of autophagy (Matsunaga et al., 2009). (3) Vesicle expansion and completion. The cytosolic form of LC3 (LC3-I) is cleaved by the cysteine protease Atg4, followed by conjugation with phosphatidylethanolamine (PE) assisted by the Atg12–Atg5–Atg16L complex, which functions as an E3–like enzyme. LC3-PE leads to PAS expansion, and cytosolic cargos are then enclosed into double membrane vesicles called autophagosomes (Geng and Klionsky, 2008). (4) Autophagosome maturation. An autophagosome matures into an autolysosome by sequential fusion with endosomes and with lysosomes, the contents of which are degraded by hydrolases therein. It is reported that autolysosome formation is related to UVRAG and expression of lysosomal-associated membrane protein 2 (Lamp-2) (Liang et al., 2008; Fortunato et al., 2009).

## Why does the RNA virus modulate autophagy?

Previous studies suggest that autophagy may be an important antiviral defense mechanism (Talloczy et al., 2006; Orvedahl et al., 2010); however, the role of autophagy in virus infection is complicated and may have opposite consequences for the viral pathogenesis. Many viruses manipulate autophagy for their own benefit by the following mechanisms.

### Forming the membrane-bounded replication compartments for viral replication, or arraying autophagic vesicles for viral particle assembly or shedding

The exploitation of autophagy has been identified in many RNA viruses including PV, CVB3, JEV, and HCV (Jackson et al., 2005; Wong et al., 2008; Tanida et al., 2009; Ke and Chen, 2011; Li et al., 2012). Increased amounts of autophagosomes, as well as colocalization of the autophagy marker protein LC3 and viral protein, were observed in virus-infected cells. In addition, cells treated with an autophagy inhibitor, or transfected with siRNA specifically obstructed autophagic processes, which reduced virus replication or virus titer. For example, in PV infection, virus yield was correlated with the induction of autophagy. Treating cells with siRNA targeting LC3 or Atg12 to block autophagy leads to reduced virus yield (Jackson et al., 2005). In addition, based on the topology of a double membrane compartment, digestion of the inner membrane under the autolysosome formation would allow efficient fusion of the autophagosomal membrane with the cytoplasm membrane. Thus, an emerging concept is that autophagy may also involve the nonlytic release of cytoplasm under autophagosome maturation, namely autophagic exit without lysis (AWOL), which may participate in the release of PV (Kirkegaard and Jackson, 2005; Taylor et al., 2009).

### Increased viral infectivity by blocking autophagic flux

Virus-induced uncompleted autophagy was reported for CVB3-, rotavirus-, and IVA- infected cells (Gannage et al., 2009; Kemball et al., 2010; Alirezaei et al., 2012; Crawford et al., 2012). In CVB3-infected pancreatic acinar cells, an increase in the number of double-membraned autophagy-like vesicles was observed upon infection. However, the accumulation of autophagy substrate p62 and the formation of large autophagy-related structures named megaphagosomes indicate that CVB3 blocks a later stage of the autophagic pathway (Kemball et al., 2010). Further results highlight the impact of autophagy on CVB3 RNA replication and translation (Alirezaei et al., 2012). It was reported that rotavirus NSP4 viroporin initiates autophagy to transport viral proteins to sites of virus replication for assembly of mature particles, which involves an increase of cytoplasmic calcium and subsequent activation of the CaMKK-β–AMPK pathway. Rotavirus also interferes with autophagy maturation; however, the mechanism is still unknown (Crawford et al., 2012). Accumulated studies reveal that M2, HA, and NS1 proteins of IAV are involved in the induction of autophagy, while only M2 has been identified as playing a critical role in impeding fusion of autophagosomes with lysosomes (Gannage et al., 2009; Sun et al., 2012; Zhirnov and Klenk, 2013).

### Escaping the host immune response

Autophagy-mediated immune responses that benefit virus replication have been reported in VSV, HCV, DENV, and JEV (Jounai et al., 2007; Ke and Chen, 2011; Jin et al., 2013). In VSV infection, the Atg5–Atg12 conjugate targets RIG-I/MDA5–MAVS-dependent type I IFN production by directly interacting with the MAVS and RIG-I, and negatively regulates MAVS-mediated NF-κB and type I IFN promoters, and permits VSV replication. Furthermore, through an unknown mechanism, HCV- or DENV-induced complete autophagy negatively regulates type I IFN production and promotes HCV replication (Ke and Chen, 2011). Recently, research about JEV has shown that in autophagy-impaired cells, virus infection induces aggregates of MAVS and activation of IFN regulatory factor 3 (IRF3), markers for activation of innate immune responses, which suggests that autophagy-mediated immune responses are required for viral replication (Jin et al., 2013).

## UPR and autophagy

As ER proliferation, which paradoxically commits the cell to cell death or survival, is observed both in UPR and autophagy, it is reasonable to propose a possible link between UPR pathways and the autophagic response. Indeed, many UPR-related transcription factors manage Atg expression (Table 1). As demonstrated previously, yeasts with mutations in the GCN2-signaling pathway are defective in starvation-induced autophagy. GCN4, which undertakes GCN2-dependent transcriptional activation, is essential for autophagy induction (Talloczy et al., 2002). Recently, results of multiple genetic models showed that the PERK–eIF2α–ATF4 pathway affects cMyc-dependent tumorigenesis by evoking cytoprotective autophagy; while pharmacologic or genetic inhibition of autophagy resulted in enhanced Myc-dependent apoptosis (Hart et al., 2012). Thus, UPR inhibition could provide new targets for the treatment of malignancies, characterized by cMyc overexpression. In addition, IRE1 also mediates autophagy in Huntington's disease under ER stress. Clearance of mutant huntingtin aggregates through autophagic flux was impaired via IRE1–TRAF2 signaling, which results in neuronal cytotoxicity (Lee et al., 2012). Although studies on UPR autophagy mainly focus on the regulation of eIF2α kinase and IRE1, transcriptional regulation of autophagic genes by ATF6 and SREBP2, a membrane-bound transcription factor activated through proteolytic processing upon ER stress, was noticed recently (Ogata et al., 2006; Seo et al., 2011; Gade et al., 2012). Death-associated protein kinase 1 (DAPK1), a positive mediator of IFN-regulated growth suppressor, is principally regulated by transcription factor C/EBP-β, one of the genes that increases expression during ER stress (Chen et al., 2004). DAPK1 promotes autophagy by phosphorylating Beclin 1, and therefore dissociating it from autophagy negative regulator Bcl2. An investigation found that activated ATF6 could directly interact with C/EBP-β carrying an ERK1/2 target site; this heterodimer then coacts to activate the DAPK1 promoter, which in turn induces autophagy. Additionally, XBP1, a downstream target of ATF6, is essential for C/EBP-β expression (Chen et al., 2004). The role of SREBP2 in autophagy was disclosed through gene ontology analysis (Seo et al., 2011). Further study shows that SREBP-2 activates autophagy gene expression, such as LC3B, ATG4B, and ATG4D, accompanied by increased LC3 puncta formation, while SREBP-2 deficiency obtains an opposite result.

**Table 1:** **Exploitation of autophagy by modulation of UPR transcription factors**.

**Transcription factor**	**Target protein**	**References**
ATF4	LC3, p62/SQSTM1, and ULK1	Milani et al., 2009; Rouschop et al., 2010; B'Chir et al., 2013; Pike et al., 2013
CHOP	ATG5, LC3, and p62/ SQSTM1	Rouschop et al., 2010; B'Chir et al., 2013; Wang et al., 2014
ATF6	DAPK1	Gade et al., 2012
C/EBPβ	DAPK1, ATG4B, and ULK1	Gade et al., 2008; Ma et al., 2011; Guo et al., 2013
SREBP2	LC3, ATG4B and ATG4D	Seo et al., 2011
XBP1	Beclin1 and Bcl2	Gomez et al., 2007; Margariti et al., 2013

In virus infection, HCV is a well-documented model illustrating UPR autophagy regulation. Induction of UPR and incomplete autophagy was observed in cells transfected with HCV JFH1 RNA. Cells treated with siRNA targeting PERK, IRE1, and ATF6 showed a suppression of LC3 conversion and a decrease of HCV RNA replication (Sir et al., 2008). In the HCV infection system, HCV induces complete autophagy and CHOP plays a leading role in UPR autophagy signaling (Ke and Chen, 2011). Further efforts to decipher how HCV activates autophagy revealed that PERK–eIF2α–ATF4 and ATF6 pathways activated CHOP expression in HCV core protein-transfected cells where the core protein had not been demonstrated to induce ER stress previously. Moreover, HCV core protein may promote ATG12 and LC3 protein expression through transcriptional control by ATF4 and CHOP, respectively (Wang et al., 2014).

Recent studies suggest that completed autophagy induced by CHIKV infection is mediated by the independent induction of the endoplasmic reticulum and oxidative stress pathways. Knockdown of IRE1 or treated cells with the ROS inhibitor *N*-acetyl-l-cysteine inhibits formation of autophagosomes as well as the conversion of LC3-I to LC3-II. Moreover, an additive inhibitory effect on autophagosome formation was observed in infected cells silenced for *IRE1*mRNA and treated with *N-acetyl-l-cysteine* (Joubert et al., 2012).

## Targeting UPR or autophagy as potential therapy in virus infection

Because UPR and autophagy play a role in viral pathogenesis, the regulation of UPR and autophagy may be an important strategy for the future development of new therapeutic approaches to combat viruses. For example, we have demonstrated that overexpression of GRP78 to relieve ER stress decreases EV71 replication (Jheng et al., 2010). Thus, agents such as GRP78/BiP inducer X (BIX) (Kudo et al., 2008) or chemical chaperone, tauroursodeoxycholic acid (TUDCA) (Ozcan et al., 2006), will be potentially useful in the treatment of EV71 (Table 2).

**Table 2:** **Compounds affecting UPR and autophagy**.

**Inhibitors/Inducers**	**Mode-of-action**	**References**
**UPR**		
GRP78/BiP inducer X (BIX)	GRP78 upregulation	Kudo et al., 2008
Tauroursodeoxycholic acid (TUDCA)	Reduces UPR	Ozcan et al., 2006
Salubrinal	Inhibitor of eIF2α dephosphorylation	Boyce et al., 2005
3,5-dibromosalicylaldehyde	Inhibits the RNase activity of IRE1αs	Volkmann et al., 2011
Sunitinib	Inhibits IRE1α *trans*-autophosphorylation, but promotes oligomerization and activates the RNase domain Inhibitor of PKR	Korennykh et al., 2009; Jha et al., 2011
STF083010	Inhibits the RNase activity of IRE1α	Papandreou et al., 2011
Nelfinavir	Induces UPR autophagy	Mahoney et al., 2013b
Sorafenib	Induces UPR autophagy	Shi et al., 2011
**AUTOPHAGY**		
Rapamycin	Induces autophagy	Ravikumar et al., 2002
Chloroquine	Inhibits autophagic flux	Yoon et al., 2010
Bafilomycin A1	Inhibits autophagic flux	Van Deurs et al., 1996
Nelfinavir	Induces UPR autophagy	Mahoney et al., 2013b
Sorafenib	Induces UPR autophagy	Shi et al., 2011
Evodiamine	Impairs autophagy	Dai et al., 2012
23-(*S*)-2-Amino-3-phenylpropanoyl-silybin	Impairs autophagy	Dai et al., 2013

There are other established strategies to inhibit viruses by modulating UPR target eIF2α phosphorylation or IRE1, e.g., salubrinal is a small molecule that prevents dephosphorylation of eIF2α and 3,5-dibromosalicylaldehyde, an IRE1 inhibitor, may cause restriction of IVA (Boyce et al., 2005; Volkmann et al., 2011).

There is emerging evidence that pharmacological agents that directly activate or deactivate autophagy influence virus replication. Evodiamine and 3-(*S*)-2-amino-3-phenylpropanoyl-silybin have been identified as anti-IVA agents aimed at multiple processes of autophagy (Dai et al., 2012, 2013). Additionally, chloroquine-suppressed HCV replication has been proved (Mizui et al., 2010).

Because UPR and autophagy are closely related, combination treatment may show a synergistic effect of their application, which was demonstrated in cancer research. The combination of nelfinavir (which induces UPR autophagy) and chloroquine enhances cytotoxicity against cancer cells (Mahoney et al., 2013b); therefore, the use of combination treatment with improved efficacy and decreased toxicity represents a promising strategy to fight viruses.

## Perspectives

Although UPR autophagy has been discussed in many research areas, its integrated response to virus infection is only now beginning to emerge. It needs to be experimentally proven whether virus-induced autophagy is associated with UPR. Furthermore, given what we know about the various means that viruses use to modulate UPR or autophagy to advantage their own virulence, the development of specific inducers or inhibitors for these molecules is one of the major challenges in this field.

### Conflict of interest statement

The authors declare that the research was conducted in the absence of any commercial or financial relationships that could be construed as a potential conflict of interest.
